# Association of Diet With Treatment Response in Dogs With Chronic Enteropathy: A Retrospective Multicenter Study

**DOI:** 10.1111/jvim.70071

**Published:** 2025-05-06

**Authors:** Sofia D. Rodrigues, Beatriz Mendoza, Maria J. Dias, Nuno S. Santos, Marine Hebert, Elisa Bettin, Francesco Signorelli, Fabio Procoli, Juan Hernandez, Rodolfo O. Leal

**Affiliations:** ^1^ Veterinary Teaching Hospital, Faculty of Veterinary Medicine University of Lisbon Lisbon Portugal; ^2^ Severn Veterinary Centre Hospital‐Worcester Worcester UK; ^3^ IVC Evidensia Hospital Veterinário Bom Jesus Braga Portugal; ^4^ CIISA, Centre for Interdisciplinary Research in Animal Health Faculty of Vet Medicine, University of Lisbon/Associate Laboratory for Animal and Veterinary Sciences (AL4AnimalS) Lisbon Portugal; ^5^ Faculty of Veterinary Medicine University of Glasgow Glasgow UK; ^6^ Oniris, Ecole Nationale Vétérinaire Agroalimentaire et de l'Alimentation Nantes France; ^7^ Anicura Ospedale Veterinario I Portoni Rossi Bologna Italy

**Keywords:** diarrhea, dogs, food management, hydrolyzed, IBD, referral

## Abstract

**Background:**

Hydrolyzed protein diets are commonly used in the first‐line approach to the treatment of dogs with naïve‐chronic enteropathy (naïve‐CE).

**Objectives:**

To characterize the responses of naïve‐CE dogs transitioned to a hydrolyzed diet and to assess the efficacy of an additional dietary trial in dogs with NRE.

**Animals:**

Eighty‐one dogs with naïve‐CE and 23 dogs with NRE.

**Methods:**

Retrospective multicenter cohort study including dogs with CE presented to three referral centers from April 2018 to December 2021. Naïve‐CE and NRE cases transitioned to hydrolyzed and alternative diets, respectively, were selected, and medical records were reviewed. Clinical response before and 4 weeks after dietary transitions (with or without concurrent therapeutic adjustments) was assessed based on stool‐consistency score or Canine Inflammatory Bowel Disease Activity Index (CIBDAI) total score in naïve and NRE cases, respectively.

**Results:**

The transition into a hydrolyzed diet was the only therapeutic change in 20% (16/81). Of these, 88% (14/16) had a decreased stool‐consistency score (*p* < 0.001). From the 23 NRE cases, the transition to an alternative diet was the sole therapeutic adjustment in 70% (16/23). Of these, the total CIBDAI score declined in 69% (11/23; *p* < 0.001).

**Conclusions and Clinical Importance:**

This study supports the need to feed a hydrolyzed diet in naïve CE cases. In cases classified as NRE, an additional transition into an alternative dietary trial seems beneficial.

**Abbreviations**
AEs: anti‐emetics
CE: chronic enteropathy
FRE: food‐responsive enteropathy
FS: fiber supplementation
GI: gastrointestinal
GPs: gastro protectants
IRE: immunomodulator‐responsive enteropathy
MrMRE: microbiome related modulation enteropathy
NRE: non‐responsive Enteropathy
PBs: probiotics

## 1 Introduction

Chronic enteropathy (CE) is a group of diseases with multifactorial origin characterized by signs of persistent or recurrent gastrointestinal (GI) disease [1], accounting for 70% of dogs presented for chronic diarrhea [2, 3]. This can be classified into food‐responsive enteropathy (FRE), microbiome‐related modulation responsive enteropathy (MrMRE), immunomodulator‐responsive enteropathy (IRE), and non‐responsive enteropathy (NRE) cases [4], depending on their response to stepwise treatment trials. If associated with low serum albumin or total protein, CE can further be subclassified as protein‐losing enteropathy [4, 5]. According to this approach, it is recommended that dietary trials are implemented as a first step. The choice is often guided by a detailed dietary history, clinical signs, and the use of either fiber‐enriched, hydrolyzed, or novel protein diets (homemade or commercial). In cases where dietary trials fail, antibiotics have historically been used as a second line approach. However, the application of novel alternatives for microbiota modulation (prebiotics, probiotics and/or fecal microbiota transplantation [FMT]) should be prioritized [4, 6]. Immunomodulators can also be considered, based on the assessment of clinical severity and progression, clinical and laboratory findings, along with histopathology, when available [4, 7]. Cases that fail to respond to this sequential approach are considered NRE cases [4, 8].

Response to dietary trials is associated with the beneficial effects on gut microbiota composition and function, on mucosal immunity, on intestinal motility, and recovery of intestinal epithelial barrier structure [9, 10]. Since proteins are considered important antigens, a dietary transition onto a new source of protein or a hydrolyzed protein diet is emphasized to reduce dietary antigenicity and intestinal inflammation [11, 12]. In addition, a diet with hydrolyzed proteins induces changes in gut microbiota composition that could be accompanied by an increase in the biotransformation of bile salts in dogs affected by CE [11]. These effects, alongside the high percentage of clinical response [3, 12, 13], justify the choice of hydrolyzed diets as the first dietary option in these cases [5, 14].

Dietary history is often incomplete and overlooked in first opinion practices [15], leading to the referral of dogs with CE without a previous hydrolyzed diet trial. This can lead to the misclassification of some dogs with FRE as suffering from NRE [4]. Considering the limitations of the dietary trials, mainly associated with food indiscretions and owners compliance, it is important to consider the possibility of an inadequate trial, especially in declared NRE cases, justifying a new strict dietary trial. Alternative strategies to manage NRE have been gathering some attention [4], data regarding further dietary transitions in these cases is still scarce.

Designed to cover the impact of diet in CE cases, this work is divided into two parts. The first part aims to identify, characterize, and monitor dogs with naïve‐CE, without previous trials with a hydrolyzed diet, referred for specialist care. The second part aims to evaluate the benefit of an additional dietary trial in dogs classified as NRE.

## 2 Material and Methods

Medical records of dogs referred for a gastroenterology consultation between April 2018 and December 2021 in three European referral centers (Veterinary Teaching Hospital—Faculty of Veterinary Medicine, University of Lisbon; Anicura Ospedale Veterinario I Portoni Rossi, Bologna and Oniris, Ecole Nationale Vétérinaire Agroalimentaire et de l'Alimentation—Nantes, France) were reviewed. Data was obtained by reviewing the gastroenterology reports performed over that specific period and selected by specific keywords (“diarrhea” and “chronic enteropathy”). Information regarding sex, age, breed, dietary history, clinical signs, medical investigation, therapies, and short‐term (four‐weeks) clinical response was collected.

Dogs included were all diagnosed with CE by a board‐certified specialist or a specialist‐in‐training. As inclusion criteria, dogs must have had a complete medical and dietary history and a follow‐up of at least 4 weeks. The diagnosis of CE was based on the presence of diarrhea for at least 3 weeks' duration with or without other concurrent GI clinical signs (vomiting, hematochezia, melena, flatulence, borborygmus, fecal tenesmus). As part of the medical work‐up, extra‐intestinal diseases (hepatic, pancreatic, adrenal or renal) and infectious causes were excluded on a case‐by‐case basis. Tests included complete blood cell count, serum biochemistry, as well as basal cortisol concentration, urinalysis, and coprology. Additional investigations included measurements of serum cobalamin concentration, as well as abdominal ultrasound scans. The clinical severity was retrospectively scored in all dogs according to either the total Canine Inflammatory Bowel Disease Activity Index (CIBDAI) or stool consistency score adapted from CIBDAI [11, 16]. Dietary history, previous use of probiotics, and fecal transplantation trials were reviewed for all the cases. Ongoing medications were recorded and divided into the following categories: supportive treatment, antibiotics, and immunomodulators. Total proteins and albumin were reviewed in all the cases to assess the possibility of protein‐losing enteropathy (PLE). Medical follow‐up was based on the medical records and owner updates.

From the study sample of CE cases, two groups were established and evaluated according to the following information.

### 2.1 Naïve‐CE Group

This group included dogs diagnosed with CE without a hydrolyzed protein diet trial before referral, which were transitioned to this diet type.

Data regarding the first referral consultation was collected, including signalment, clinical signs, dietary history, and concurrent medication, along with albumin and total protein levels. Cases were assessed according to diet change and concurrent treatments (no change on current treatment vs. current treatment adjusted). The decision to add/change medication was made case by each clinician.

Clinical response was assessed based on the variation of stool consistency, adapted from the total CIBDAI score [11, 16]. This was assessed during the first consultation and extrapolated from the owner's feedback by phone, e‐mail, or clinician reports at least 4 weeks after the initial consultation. Stool consistency score was graded as follows: 0 (normal), 1 (slightly soft feces), 2 (very soft feces) and 3 (watery diarrhea) [11]. Clinical improvement was judged based on a decrease in the stool consistency score between the two time points.

### 2.2 NRE Group

Dogs were considered NRE if they failed to respond to a previous hydrolyzed dietary trial, microbiota‐related strategies, and immunomodulator trials. This group included dogs classified as NRE, who were transitioned into an alternative new diet after that classification. Cases were retrospectively reviewed for signalment, clinical signs, dietary history, and medical treatment changes, along with the albumin and total protein levels. Concurrent treatment modifications were at the clinician's discretion and were non‐standardized. These cases were transitioned into a novel hydrolyzed diet, a commercial novel protein diet, a homemade novel protein source diet, a commercial fiber‐enriched diet, or a commercial highly digestible non‐hydrolyzed diet, and information regarding these diets was recorded.

Clinical response was evaluated 4 weeks after this transition using data obtained from medical records and by evaluating the CIBDAI score progression (before and after the new dietary trial). CIBDAI score was classified as: insignificant (0–3), mild (score 4–5), moderate (score 6–8) and severe (score 9 or greater) [11, 16].

### 2.3 Statistical Analysis

Data was collected, uploaded, and analyzed using Microsoft Excel 2022 software and SPSS (IBM SPSS statistics version 28.0.1.0). Descriptive statistics were used, and the results were reported as absolute numbers and percentages (%). Numerical variables were assessed for normality using the Shapiro–Wilk test. Median and interquartile range (25th‐75th) or mean ± standard deviation were used if case distribution was non‐normal or normal, respectively. Comparison of fecal scores and CIBDAI scores was assessed using the Wilcoxon pairwise‐signed rank test for a significance value set as *p* < 0.05 (confidence interval of 95%).

## 3 Results

A total of 104 dogs with CE were initially selected. Of these, 77.9% (81/104) had not undergone a hydrolyzed diet trial before referral and were transitioned to one, being included in the “naïve‐CE group” From the same study sample, 22.1% (23/104) were classified as NRE and were started on a new diet, being included in the “NRE group”

### 3.1 Naïve‐CE Group

The median age of this group was 6 (3–8.2) years old. 65% (53/81) were male and 35% (28/81) female. A total of 78% (63/81) were pure‐bred dogs. Labrador Retriever and German Shepherd were the most frequent breeds (11% (9/81) and 10% (8/81), respectively). The clinical presentation was characterized by diarrhea in all cases (81/81). Other clinical signs included vomiting in 57% (46/81), hematochezia (24% [19/81]), melena (7% [6/81]), fecal tenesmus (7% [6/81]), borborygmus (6% [5/81]) and flatulence (1% [1/81]). Total protein and albumin levels were available in 88% (71/81) of the cases. These parameters were both normal in 78% (55/71) of cases. Panhypoproteinemia was present in 14% (10/71), hypoalbuminemia with normal total proteins in 4% (3/71) and hypoproteinemia with normal albumin in 4% (3/71).

At presentation, 47% (38/81) were not on any medication; 30% (24/81) were on supportive therapy (8/24 on gastro‐protectants (GPs—proton‐pomp inhibitors, histamine‐type 2 receptors or sucralfate), 3/24 on anti‐emetics (AEs), 12/24 on probiotics (PBs), 2/24 on cobalamin supplementation, 1/24 with psyllium and 1/24 with lactulose); 12% (10/81) were receiving antibiotics (4/10 on metronidazole, 2/10 on a combination of metronidazole with spiramycin, 1/10 on cephalexin, 1/10 on cefovecin, 1/10 on enrofloxacin and 1/10 with amoxicillin‐clavulanic acid), and 11% (9/81) were on immunomodulators at the time of presentation (eight in nine under prednisolone [0.5–1 mg/kg q12h] and one in nine with prednisolone combined with cyclosporine [5 mg/kg q12h]).

**FIGURE 1:**
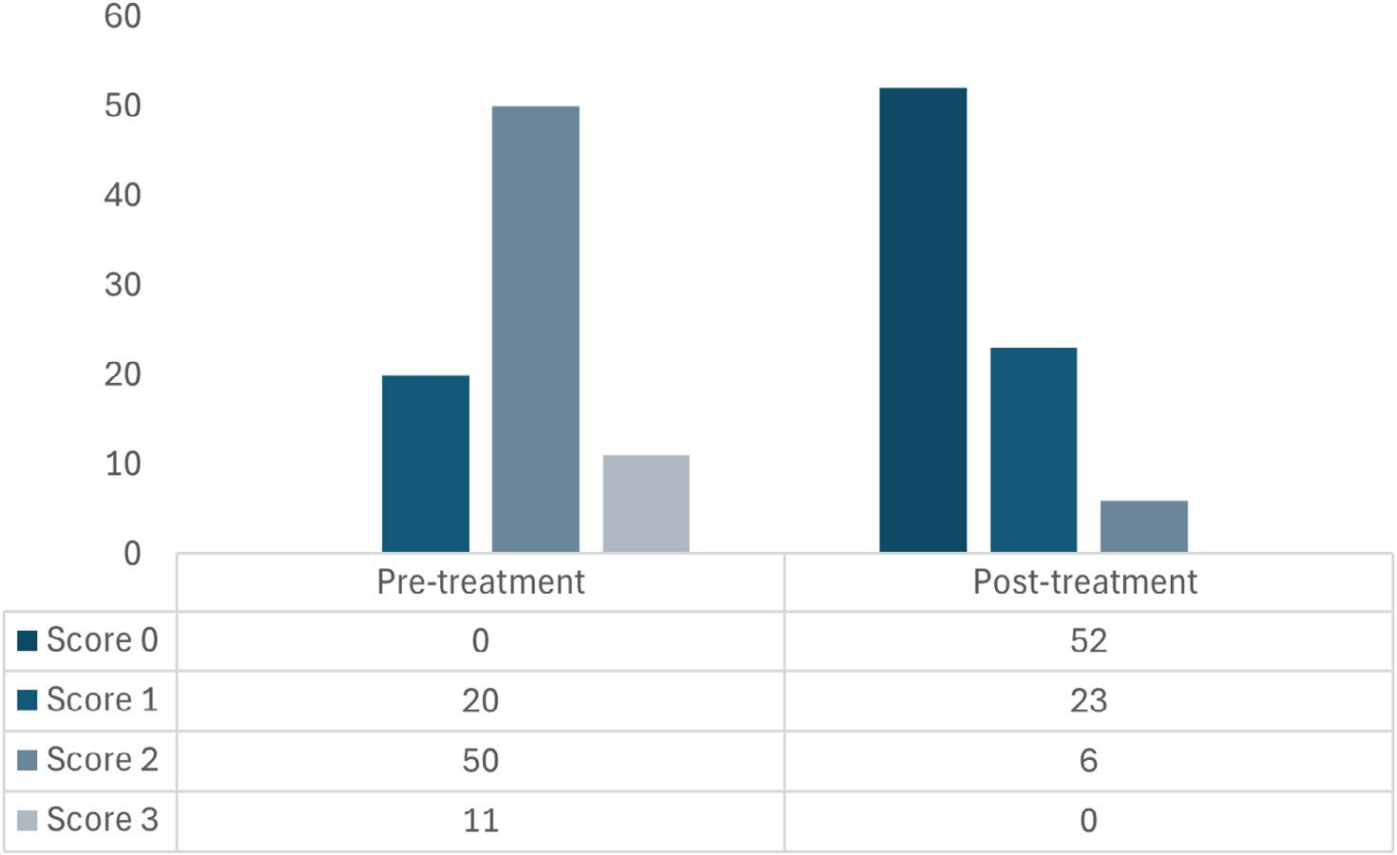
Overall stool consistency score variation, before and 4 weeks after treatment in dogs with naïve‐CE (*n* = 81) transitioned into a hydrolyzed diet (with or without concurrent therapeutic adjustments).

All 81 dogs (100%) were started on a hydrolyzed diet trial after initial presentation. Of these, 90% (73/81) of dogs were changed to a soy‐based hydrolyzed protein diet, and the remaining 10% (8/81) were fed a chicken‐based hydrolyzed diet (Table 1). Four weeks after dietary transition (with or without concurrent medical adjustments), stool consistency score improved in 95% (77/81) of patients and remained the same in 5% (4/81). Detailed variation in stool consistency score is described in Figure 1. Overall, the stool consistency score improved (*p* < 0.001), having decreased from a median of 2 (2) to 0 (0–1) after dietary intervention.

**TABLE 1:** Types of hydrolyzed diets to which dogs with naïve CE were transitioned (*n* = 81).

Nutrition and analytical constituents (g/100 kcal)	Commercial hydrolyzed diets			
	Soy‐based (*n* = 73)		Chicken‐based (*n* = 8)	
	Brand A (*n* = 67)	Brand B (*n* = 6)	Brand C (*n* = 5)	Brand D (*n* = 3)
Protein	130	136 (min)	133	161
Fat content	252	152 (min)	267	197
Crude fiber	1374	864 (max)	1466	436
ME (g/100 kal)	27.4	3855	341	3534
ME (kcal/100 g)	364	386	341	353

**FIGURE 2:**
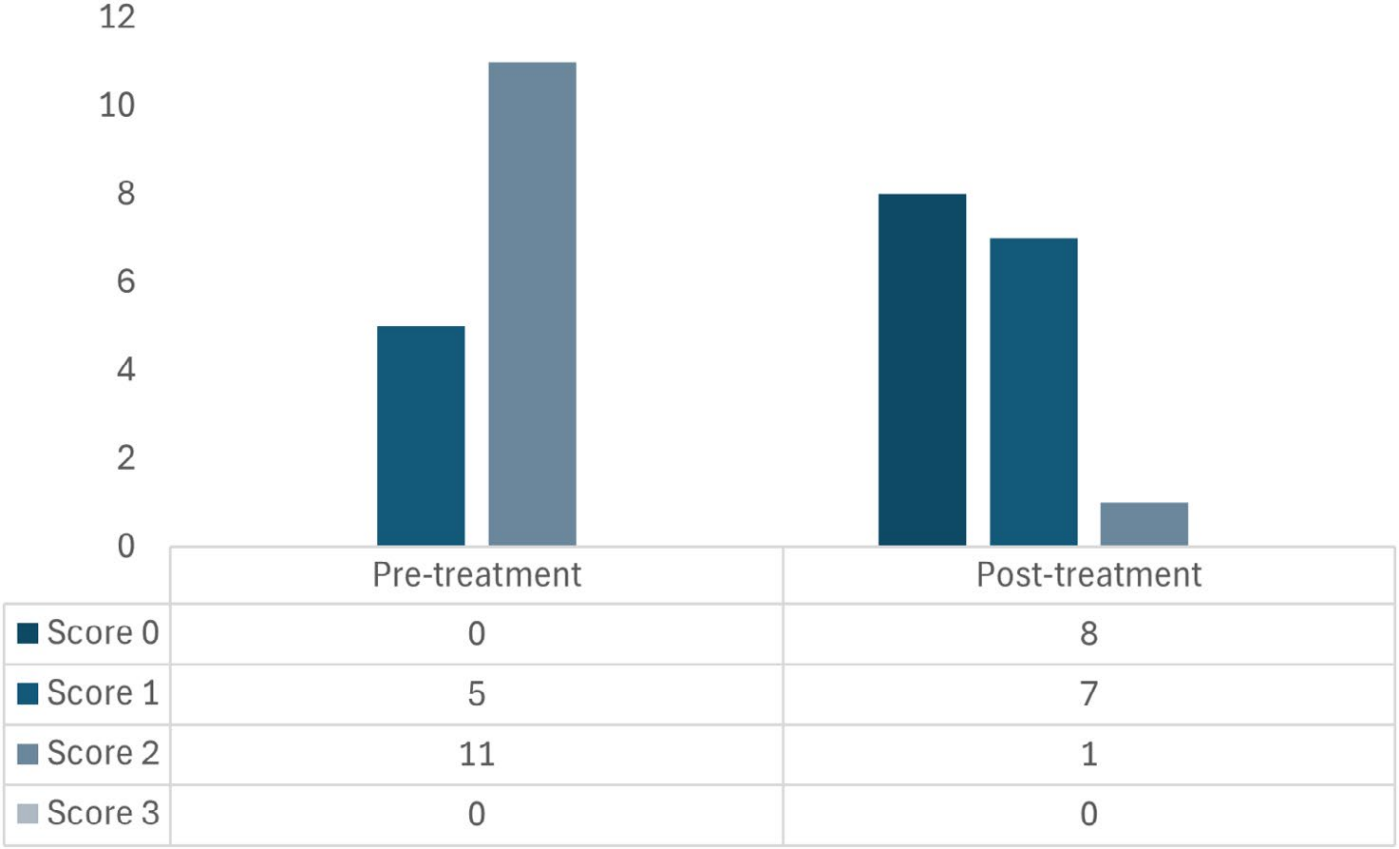
Stool consistency score variation, before and 4 weeks after treatment in dogs with naïve‐CE (*n* = 16) in which dietary change was the only treatment intervention.

Transition to a hydrolyzed diet was the only treatment change in 20% (16/81) of dogs. Of these (*n* = 16), four dogs continued without any medication, while previous treatments were continued for the other 12 dogs, at the same dosages and frequencies (four on supportive treatment, three on antibiotics, and five on immunomodulators). All these cases had normal total protein and albumin levels. In these 16 dogs, improvement in stool consistency score was noted in 88% (14/16), while no change was noted in 13% (2/16). Overall, median stool consistency decreased from 2 (1‐2) to 0.5 (0–1; *p* < 0.001). Detailed variation of stool consistency score is described in Figure 2.

In the remaining 80% (65/81) cases, the new hydrolyzed diet was started along with other therapeutic changes. In 62% (40/65) of these cases, supportive therapy was added or changed (27/40 on PBs, 17/40 with cobalamin supplementation, 10/20 on AEs, 8/40 on GPs, and 6/40 with psyllium); in 19% (12/65) antibiotics were added (metronidazole in 10/12, enrofloxacin in 1/12, doxycycline in one, and a combination of sulfasalazine and metronidazole in another one). Immunomodulator treatment was started in 20% (13/65) of cases, with prednisolone (0.5‐1 mg/kg q24h) started in 12/13 and chlorambucil (initial dosage of 4 mg/m2 q24h followed by a decrease to 2 mg/m2 q24h) in 1/13 cases. In these 65 cases, the median stool consistency score decreased from 2 (2) to 0 (0–1) after medical and dietary intervention (*p* < 0.001).

### 3.2 NRE Group

Median age of cases included in this group was 6 (4–10) years old; 57% (13/23) were male and 44% (10/23) were female. Pure‐bred dogs were overrepresented, accounting for 83% (19/23) of the cases. The clinical presentation was characterized by diarrhea in all cases (23/23). Other noted clinical signs included vomiting in 52% (12/23), hematochezia (26% [6/23]), borborygmus (17% [4/23]), flatulence (9% [2/23]) and melena (4% [1/23]). Total protein and albumin levels were available in 87% (20/23) of the cases. Of these, both parameters were normal in 55% (11/20), 30% (6/20) had hypoproteinemia and hypoalbuminemia, and 15% (3/20) had hypoproteinemia with normal albumin. In this group, a histologic diagnosis was available for 52% (12/23) of cases, all consistent with chronic lymphoplasmacytic enteritis. The remaining 48% (11/23) dogs had no histopathology information in medical records.

**FIGURE 3:**
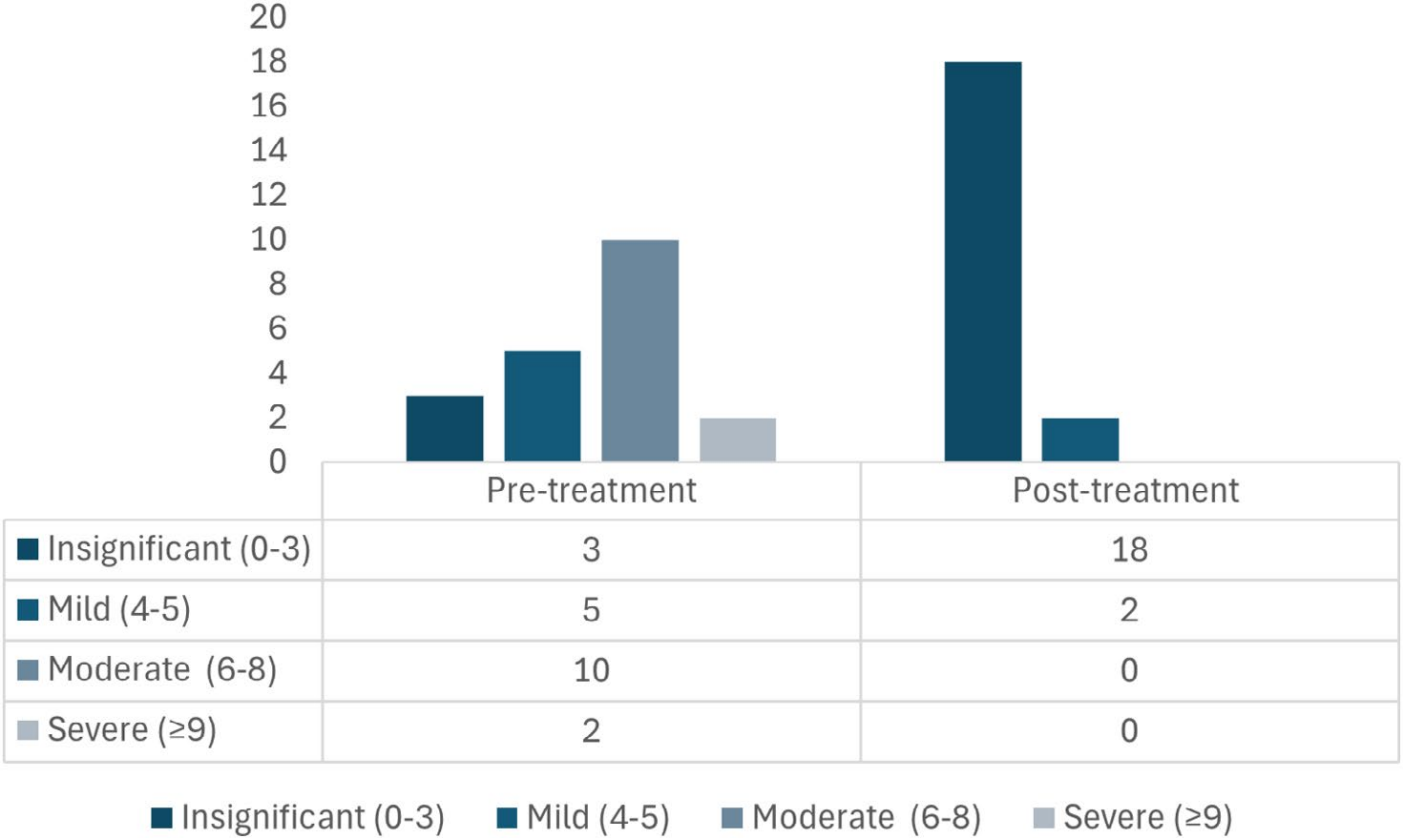
Overall total CIBDAI variation, before and 4 weeks after treatment in dogs with NRE (*n* = 23) transitioned into an alternative diet (with or without concurrent therapeutic adjustments).

The new dietary transition was implemented with different diet types, with 70% (16/23) of dogs transitioned to a different commercial hydrolyzed diet, 17% (4/23) to a homemade novel protein diet, and one each (4% [1/23]) transitioned to a commercial novel protein, commercial fiber‐enriched, and commercial highly digestible non‐hydrolyzed diet (Table 2). Three cases were lost to follow‐up, and so CIBDAI post‐treatment was not assessed. Overall, 90% (18/20) of NRE cases showed improvement in CIBDAI after dietary transition with or without concurrent medical treatment adjustments, while no change was noted in the remaining 10% (2/20). Total CIBDAI improved from a median of 6 (4–7) to 3 (0–3.8; *p* < 0.01). Total CIBDAI pre‐and post‐treatment variation is presented in Figure 3.

**TABLE 2:** Types of alternative diets to which dogs with NRE were transitioned (*n* = 23).

Nutrition and analytical constituents (%)	Commercial hydrolyzed diets (*n* = 16)			Commercial novel protein source (*n* = 1)	Commercial fiber‐enriched (*n* = 1)	Commercial highly digestible GI diet non‐hydrolyzed (*n* = 1)	Homemade novel protein (*n* = 3)	
	Soy‐based (*n* = 10)	Chicken‐based (*n* = 4)	Feather‐based (*n* = 2)	Duck‐based (*n* = 1)	Chicken based (*n* = 1)	Poultry‐based (*n* = 1)	Duck + sweet potato (*n* = 2)	Turkey + green beans + potato (*n* = 1)
Protein	130	161	148	95 (min)	126	127	N/A	N/A
Fat content	252	197	161	196 (min)	204	265		
Crude fiber	1374	436	1330	587 (max)	384	1215		
ME (kcal/kg)	3640	3534	3758	3405	3430	3427		
ME (kcal/100 g)	364	353	376	341	343	343		

The implementation of an alternative diet was the sole treatment change in 70% (16/23) of dogs with NRE. Of these, 69% (11/16) were transitioned to another hydrolyzed diet, 13% (2/16) to a home‐made diet, 6% (1/16) to a fiber‐enriched diet, 6% (1/16) to a commercial novel protein diet, and lastly, 6% (1/16) to a commercial highly digestible non‐hydrolyzed diet. These cases were already on immunomodulators, and treatment was continued at the same dosages and frequencies (10/16 on prednisolone [0.5–1 mg/kg q12h]); 1/16 on cyclosporine ([5 mg/kg q24h]) and 5/16 on a combination of both drugs. Antibiotic and anti‐inflammatory treatments were also continued as previously prescribed in 3/16 dogs, with 2/16 on metronidazole and 1/16 on sulfasalazine, respectively.

**FIGURE 4:**
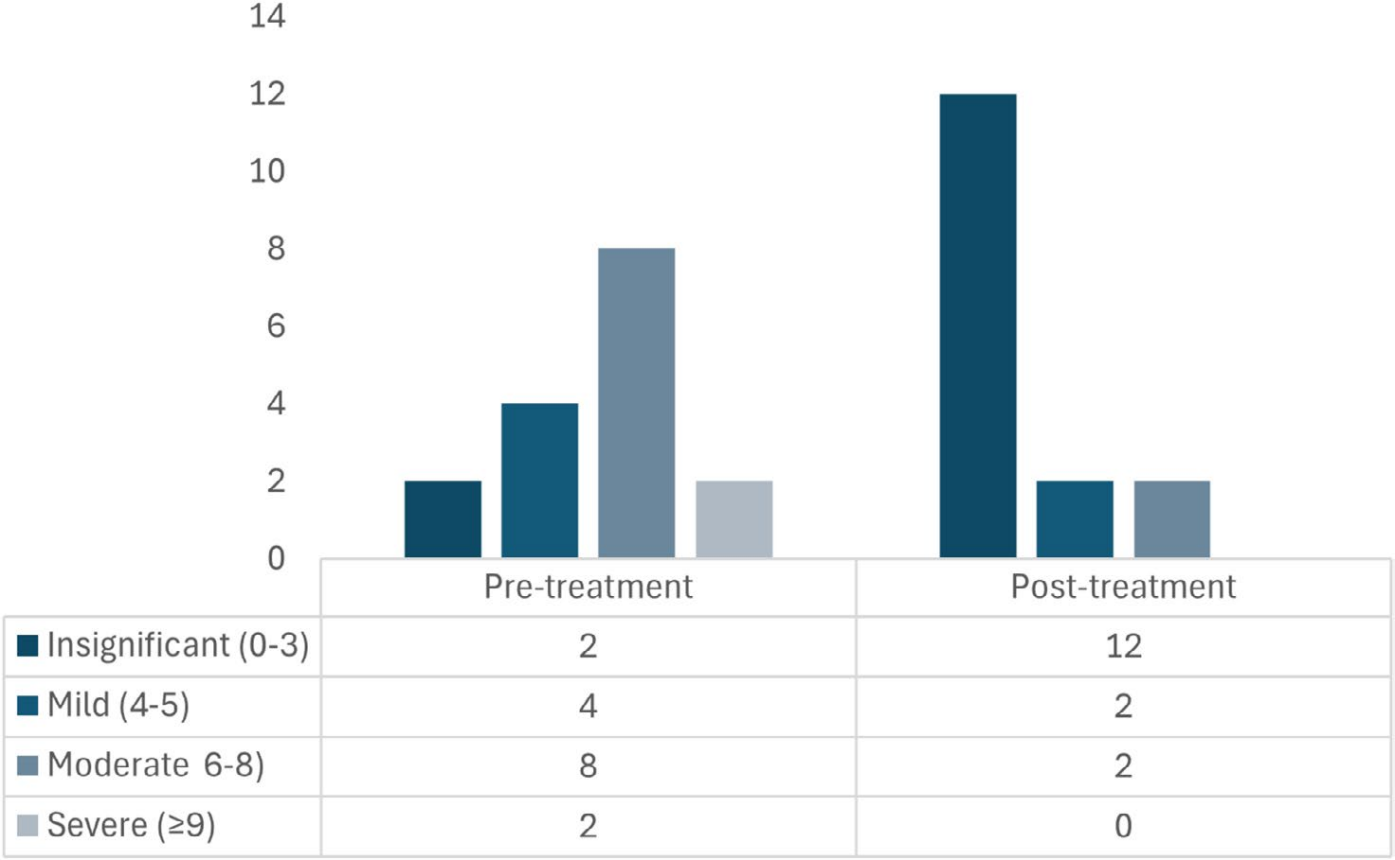
Total CIBDAI variation, before and 4 weeks after treatment in dogs with NRE (*n* = 16) in which dietary change was the only treatment intervention.

Total CIBDAI score was retrospectively calculated before and after dietary transition in the 16 dogs of this group (Figure 4). CIBDAI decreased in 69% (11/16) of cases, while no change was noted in 31% (5/16). Median pre‐diet and post‐diet adjustment CIBDAI decreased (*p* < 0.001) from 6 (4–7) to 3 (0.5–5). Of the dogs with improvement noted on the CIBDAI, eight were changed to another commercial hydrolyzed diet, one to homemade diet, another to a commercial fiber‐enriched diet, and the other to a commercial novel protein diet.

In 30% (7/23) of NRE, the diet was changed along with changes in the medical treatment. Of these, five in seven transitioned to another hydrolyzed diet, whereas the remaining two transitioned into a homemade diet. Regarding medical treatment, three in seven had changes in supportive therapy (one started cobalamin supplementation, one psyllium and another FMT); three in seven had changes in immunomodulation therapy (one dog changed from azathioprine to prednisolone and cyclosporine and another two from prednisolone to cyclosporine) and in one in seven, antibiotics were changed (cefovecin to spiramycin). Post‐treatment CIBDAI data was available for only four cases in this group, with the other three cases lost to follow‐up. Of these, one maintained the same score while the other three decreased CIBDAI scores to clinically irrelevant values.

## 4 Discussion

This study highlights the importance of dietary management in two subsets of CE cases: Cases without previous trials of feeding a hydrolyzed diet and cases previously classified as NRE.

In our study sample, more than half of CE cases seen for referral consults did not have a prior hydrolyzed diet trial, with a relevant percentage (11%–13%) of these cases already receiving antibiotics and/or immunomodulators at the time of referral. It is uncertain whether it was previously suggested to the owners, but declined, or not recommended; however, this highlights a lack of consistency and a standardized approach to the dietary management of this population.

All these cases were started on a hydrolyzed diet after the initial consultation at a referral level. This agrees with a recently proposed scheme regarding the dietary approach to canine CE, stating that hydrolyzed diets are the leading choice in non‐responsive cases compared to other diets, such as fiber‐enriched, highly digestible, and GI low‐fat diets [17].

Focusing on dogs with naïve‐CE, signalment findings were in line with previous studies focusing on food‐responsive enteropathy cases [18]. All cases presented with diarrhea, as per the inclusion criteria; however, other GI signs were also reported, highlighting the varied presentation these cases might have. Most dogs had normal total protein and albumin levels, showing that cases of PLE were not frequent in this study sample. In this study, all the cases with dietary transition as the only therapeutic choice had normal total protein and albumin levels, while those with hypoproteinemia/hypoalbuminemia had concurrent dietary and medical adjustments. However, dietary management alone, without added medical therapy, can be considered in potential PLE cases that are overall clinically stable [2, 18].

All dogs of the naïve‐CE group transitioned to a hydrolyzed diet. A soy‐based diet was the most common choice, likely because of its hydrolyzed protein source and possible immune modulation benefits [9].

Overall, in almost all cases, with or without therapeutic adjustments besides diet, there was an improvement in stool consistency score, with about 65% of dogs showing normal stools 4 weeks after medical consultation. These findings highlight the number and overall proportion of CE dogs responding clinically to dietary intervention [19].

In about 20% of naïve‐CE cases, diet change was the only therapeutic intervention. This group allowed us to evaluate the direct effects of diet with no changes to the ongoing treatments, with most of these cases improving stool consistency, showing the relevance of diet as an early therapeutic option in CE cases.

Along with dietary change, a considerable proportion of cases received additional treatments, reflecting the case‐by‐case individual management needed in practice. Owners tend to have high expectations, favoring the use of complementary drug therapies and multimodal approaches [20]. In these cases, PBs were the most frequently administered treatment, potentially justified by their theoretical benefits in antagonizing undesired microbial species and enhancing the modulation of the immune and intestinal systems [21]. However, despite their perceived utility, the evidence base supporting their use is scarce [21]. A recent review highlighted that, particularly in chronic gastrointestinal diseases, probiotics yielded no additional benefits when compared to dietary management alone, emphasizing the key role of diet on the clinical outcome of these dogs [21, 22]. In a smaller percentage of cases, antibiotics were added possibly due to their historical immunomodulatory properties or due to an individual case‐by‐case suspicion of infectious origin [2]. With increasing evidence supporting alternative strategies of microbiota modulation, the use of antibiotics is now debatable [4]. This study includes cases managed before the advent of these new strategies and protocols, and the results might in part reflect the lack of routine use of these procedures in the referral setting at the time. In about 20% of cases, immunomodulators were started in conjunction with diet. This decision was made by the leading clinician on a case‐by‐case basis and was possibly made based on the severity and duration of clinical signs and impact on the patient's quality of life, along with the possible need to improve the owner's compliance. In this subset of cases, in which dietary intervention was combined with concurrent therapeutic changes, stool consistency score also improved, reflecting the multimodal approach often conducted in practice.

Regarding NRE cases, signalment findings were in line with previous studies, although recognizing that age and breed overlap among different subtypes of CE [2, 4, 18]. In a similar way to the naïve‐CE group, apart from chronic diarrhea, other signs were also noted. Over half of NRE cases had normal total proteins and albumin levels, excluding ongoing PLE. Histopathology results were only available in half of the cases. Intestinal biopsies are considered relevant in NRE cases; however, due to the owner's financial restrictions and perceived anesthetic risk, these aren't consistently obtained in these cases. In the authors' opinion, this highlights that, even in a referral setting, gastrointestinal sampling might be declined by owners, with the clinician needing to prioritize a probabilistic approach. Considering the lack of histologic samples in some of these cases, diffuse neoplasia or occult infection could not be formally excluded in these cases. However, considering the CE classification based on therapeutic response, the lack of histologic data, in most cases, does not seem to have a direct impact on the results.

Considering the entire subset of NRE cases, an alternative dietary change induced a notable improvement in total CIBDAI score in 90% of dogs. To the best of the authors' knowledge, there is currently a lack of specific research addressing the specific role of diet as a rescue treatment in cases of canine NRE [23]. Nevertheless, there is a growing recognition of the pivotal role of diet within multimodal treatment protocols, representing a universally applicable therapeutic adjustment across all dogs afflicted with CE [24]. Notably, board‐certified specialists consistently rethink dietary management, with or without other medical adjustments, even when a previous dietary trial was unsuccessful. Detailing these cases of NRE, dietary intervention was the exclusive therapeutic adjustment in more than two‐thirds of cases. This observation implies awareness regarding the benefits of diet, but also the potential side‐effects of other treatment options, which might affect the patient's quality of life, without clear additional benefits [21]. Over two‐thirds of cases were transitioned into an alternative hydrolyzed diet, while the remaining were transitioned to home‐made or alternative diets (including fiber‐rich and highly digestible options). Along with considerations regarding the main protein in the diets used, other differences in nutritional composition can affect the patient's clinical response and influence the clinician's choice. Although diet transitions were established on a case‐by‐case basis, the overall preference for an alternative hydrolyzed diet was likely due to the already known benefits of these diets, practicality, easy compliance, or clinician preferences. More than the direct decrease in antigenicity, as the benefit of dietary transitions is not always linked to their direct immune effect, the effect of different protein sources and diet components on the gut microbiota, which can also be achieved with home‐made diets, can lead to improvement of the clinical signs [4, 25]. In this subset of cases, CIBDAI score improved after dietary transition. Since the transition into an alternative diet was the only therapeutic adjustment, this improvement highlights the role of reassessing diet in cases which can be misclassified into NRE and are, in fact, difficult FRE cases in which additional dietary modifications are still warranted [4, 26]. These results also align with other studies that demonstrated a notable influence of diet on the clinical outcomes of dogs undergoing immunomodulation treatments [23, 27].

In about one third of NRE cases, treatment adjustment included a dietary transition to an alternative diet and concurrent medical changes, including supportive treatment or immunomodulation adjustments. Due to the lack of follow‐up of these cases, CIBDAI comparisons were not performed and it is not possible to draw conclusions regarding this group as a whole; however, we would expect that the results of this group would be similar to the other groups and in agreement with recent literature indicating dietary transition is a relevant point in the multimodal approach of NRE cases [27].

As a retrospective cohort study, these conclusions are impacted by various limitations. The assessment of clinical improvement was based on stool consistency and total CIBDAI score, predominantly relying on the owner's subjective evaluation and medical records. Therefore, although we tried to compare objective data, there is some unavoidable degree of subjectivity that might influence these results. Nonetheless, it is noteworthy that a previous study identified an unexpectedly correlation between follow‐up occurrences and the perceived clinical outcome from the owner's perspective, supporting that owner's reports, albeit subjective, are still a reasonable tool to judge clinical improvement [28]. In dogs with naïve‐CE, the available data did not allow for an accurate assessment of the total CIBDAI score. After team reflection, authors selected the fecal consistency score since it was the most consistent information available in these cases. While recognizing the degree of subjectivity associated with the lack of standardization and the use of information based on owners' feedback, the authors believe that the assessment of this score minimized this limitation. Some patients included in our study were hypoproteinemic/hypoalbuminemic. Due to the lack of consistent investigation and follow‐up of these cases, it is possible that some PLE cases were included in both groups of this study.

A small percentage of cases did not improve in stool consistency, nor CIBDAI. The lack of improvement with diet or combined therapies could have been due to the inclusion of cases with intestinal neoplasia, other GI diseases, or extra gastrointestinal diseases, which might have been overlooked. Another crucial point is the owners' compliance with a restricted diet regimen or medications, which could have influenced the outcome of these cases.

In both study groups, most cases showed improvement in fecal consistency in response to the introduction of the new diet, whether as the single change or in combination with other therapeutic changes. However, it should be noted that several treatments were already ongoing or were prescribed concurrently to the diet, limiting interpretation as to what proportion of the clinical improvement was attributable to the diet alone. It is plausible that clients prepared to pursue referral are more receptive to following a strict diet trial, which could lead to a higher success rate with dietary management after referral [19]. Nonetheless, even recognizing this limitation, these results reinforce the relevance of diet in the multi‐modal approach of CE cases.

## 5 Conclusion

This study emphasizes the importance of diet in the management of CE cases, not only as a first therapeutic approach but also in NRE cases. As an early therapeutic intervention, a transition to a hydrolyzed diet without additional changes induced a relevant improvement in stool consistency in almost all cases of naïve‐CE. In NRE cases, a transition to an alternative diet (either hydrolyzed, home‐made or highly digestible option) as a single therapeutic change induced a remarkable improvement in CIBDAI in more than two‐thirds of cases in our study. These results highlight that dietary management is an essential component in the medical approach to CE cases, whether alone or in combination with concurrent treatments.

## Disclosure

Authors declare no off‐label use of antimicrobials.

## Ethics Statement

Authors declare no Institutional Animal Care and Use Committee or other approval was needed. Authors declare human ethics approval was not needed.

## Conflicts of Interest

Fabio Procoli, Juan Hernandez and Rodolfo Oliveira Leal have lectured for Royal Canin, Hills, Purina and Dechra over the past years in the field of veterinary gastroenterology. None of these companies had influence in this study. The other authors declare no conflicts of interest.
