# Erratum for “Association of Diet with Treatment Response in Dogs With Chronic Enteropathy: A Retrospective Multicenter Study”

**DOI:** 10.1111/jvim.70153

**Published:** 2025-06-18

Rodrigues SD , Mendoza B , Dias MJ , Santos NS , Hebert M , Bettin E , Signorelli F , Procoli F , Hernandez J , Leal RO , Association of Diet With Treatment Response in Dogs With Chronic Enteropathy: A Retrospective Multicenter Study. J Vet Intern Med. 2025 May‐Jun;39(3):e70071. doi: 10.1111/jvim.70071 PMC1205387440326642 PMID: 40326642; PMCID: PMC12053874.

In the above‐mentioned article, details concerning nutrition and analytical constituents of commercial diets are incorrectly presented in Tables 1 and 2. Correct tables are below.

**TABLE 1:** Types of hydrolyzed diets to which dogs with naïve CE were transitioned (*n* = 81).

Nutrition & Analytical Constituents	COMMERCIAL HYDROLYZED DIETS			
	SOY‐BASED (n=78)			CHICKEN‐BASED (n=3)
	BRAND A (n=67)	BRAND B (n=6)	BRAND C (n=5)	BRAND D (n=3)
Protein (g/100Kcal)	5.8	4.9	6.4	5.0
Fat content (g/100Kcal)	3.0	4.4	3.21	4.0
Crude fiber (g/100Kcal)	0.6	0.8	0.6	1.8
ME (kcal/100g)	364	386	341	353

**TABLE 2:** Types of alternative diets to which dogs with NRE were transitioned (*n* = 23).

Nutrition & Analytical Constituents	COMMERCIAL HYDROLYZED DIETS (n=16)			COMMERCIAL NOVEL PROTEIN SOURCE (n=1)	COMMERCIAL FIBER‐ENRICHED (n=1)	COMMERCIAL HIGHLY DIGESTIBLE GI DIET NON‐HYDROLYZED (n=1)	HOMEMADE NOVEL PROTEIN (n=3)	
	SOY‐BASED (n=10)	CHICKEN‐BASED (n=4)	FEATHER‐BASED (n=2)	DUCK‐BASED (n=1)	CHICKEN BASED (n=1)	POULTRY‐BASED (n=1)	Duck+sweet potato (n=2)	Turkey+green beans+potato (n=1)
Protein (g/100Kcal)	5.8	5.0	4.8	9.0	6.8	6.7	N/A	N/A
Fat content (g/100Kcal)	3.0	4.0	4.4	4.4	4.2	3.2	N/A	N/A
Crude fiber (g/100Kcal)	0.6	1.8	0.5	1.5	2.0	0.7	N/A	N/A
ME kcal/100g	364	353	376	341	343	343	N/A	N/A

In Table 1, Brand C main source of protein is not chicken‐based but soy‐based. This was also incorrect in the text (section 3 Results; 3.1 Naïve‐CE Group) where it is written in second paragraph, second sentence “Of these, 90% (73/81) of dogs were changed to a soy‐based hydrolyzed protein diet, and the remaining 10% (8/81) were fed a chicken‐based hydrolyzed diet”. Data is corrected in the tables and the sentence should be as follow: “Of these, **96% (78/81)** of dogs were changed to a soy‐based hydrolyzed protein diet, and the remaining **4% (3/81)** were fed a chicken‐based hydrolyzed diet.”

We do apologize for these errors.

